# Beyond the Threatened Limb: Acute Limb Ischemia as a Diagnostic Attractor

**DOI:** 10.7759/cureus.116882

**Published:** 2026-09-24

**Authors:** Antoine Smith, Soheil Zahir

**Affiliations:** 1 Emergency Department, Centre Hospitalier Régional Sambre et Meuse, Sambreville, BEL

**Keywords:** acute limb ischemia, atrial fibrillation, cancer-associated thrombosis, diagnostic reasoning, low-flow state, multimorbidity, point-of-care ultrasound, rutherford iia

## Abstract

Acute limb ischemia is a time-sensitive vascular emergency, but in older patients with active cancer and multimorbidity, the threatened limb can become a diagnostic attractor: a correct specialized diagnosis that narrows attention before whole-patient physiology is integrated. We report an 81-year-old man with metastatic cecal adenocarcinoma receiving folinic acid, fluorouracil, and oxaliplatin (FOLFOX) who presented on day one with three days of progressive left lower limb claudication, coldness, discoloration, absent pulses, preserved motor and sensory function, and no rest pain. The presentation was clinically compatible with Rutherford I-IIa acute limb ischemia; bedside Doppler signals were not documented, precluding a definitive retrospective distinction between these categories. Concurrent findings included new atrial fibrillation, basal crackles, peripheral edema, markedly elevated N-terminal pro-B-type natriuretic peptide and troponin, bilateral pleural effusions, and radiological concern for low cardiac output. Computed tomography angiography (CTA) showed severe distal superficial femoral artery stenosis, long popliteal occlusion, slow distal flow, and limited runoff, favoring an acute-on-chronic ischemic process. On day two, a 6 mm x 60 mm covered stent was placed across the distal superficial femoral artery lesion. The treated lesion had a satisfactory technical result, but distal runoff remained severely compromised. Therapeutic anticoagulation initiated on day one was not maintained; discharge treatment on day three included prophylactic-dose enoxaparin and aspirin, with no rationale for the change documented in the available record. He returned on day five with recurrent coldness and toe necrosis despite normal lactate. No repeat vascular imaging was available to define the mechanism. Care became palliative on day 13, and he died on day 58. This case illustrates that a correct vascular diagnosis should not terminate integrative emergency reasoning. We propose that a vascular-plus-physiology cross-check may help preserve attention to systemic physiology and goals of care, but it is an educational framework rather than a validated clinical algorithm.

## Introduction

Acute limb ischemia is usually taught and managed as a limb-centered emergency. This is appropriate: delayed recognition of a threatened limb can lead to tissue loss, reperfusion injury, compartment syndrome, limb loss, or death. The Rutherford classification remains a practical bedside framework for identifying whether a limb is viable, marginally threatened, immediately threatened, or irreversibly damaged [1,2].

Emergency physicians, however, rarely evaluate an isolated limb. The airway, breathing, circulation, disability, and exposure (ABCDE) approach provides a structured framework for rapid whole-patient assessment and prioritization of immediately life-threatening problems [3]. Patients with active malignancy are also at increased risk of thrombotic events because of cancer-associated hypercoagulability and treatment-related factors [4,5], providing an important link between malignancy and acute vascular presentations such as acute limb ischemia. In patients who are older, multimorbid, or receiving active oncological treatment, the most visible anatomical lesion may become a cognitive and organizational attractor. This may promote anchoring and premature closure, particularly once a plausible specialty diagnosis has been established [6-8]. Multimorbidity further complicates this process because single-disease pathways may not capture interacting physiological risks [9,10].

We report the case of an older man with metastatic colorectal cancer, new atrial fibrillation, clinical congestion, cardiac biomarker elevation, and radiological concern for low cardiac output who presented with acute limb ischemia. The limb required urgent vascular assessment. The central question is how emergency physicians can activate a specialized vascular pathway without allowing that pathway to end whole-patient physiological reasoning.

## Case presentation

Baseline context

The patient was an 81-year-old man living at home with his wife and was described as still autonomous at the time of presentation. No palliative limitation of care had been documented. Approximately two months before day one, he had been diagnosed with moderately differentiated cecal adenocarcinoma, metastatic at diagnosis with hepatic and peritoneal involvement (cT3N0M1c). He was receiving systemic treatment with folinic acid, fluorouracil, and oxaliplatin (FOLFOX); the most recent treatment cycle had been administered nine days before day one.

His medical history included hypertrophic cardiomyopathy, osteoarthritis, scapulohumeral periarthritis, varicose vein disease, and complex regional pain syndrome. His surgical history was extensive and included left Achilles tendon repair, varicose vein surgery, left meniscal surgery, bilateral total knee arthroplasties, prostatectomy followed by radiotherapy, gastrectomy for massive hemorrhage complicated by acute respiratory distress syndrome, acute kidney injury, and septic shock, incisional hernia repair, and surgery for bilateral retinal detachment. He was a former smoker. No drug allergy was known.

Emergency presentation and initial assessment

On day one, he presented with a three-day history of rapidly progressive left lower limb claudication, coldness, and discoloration. He did not report rest pain. He also denied chest pain, perceived palpitations, dyspnea, cough, fever, chills, or sweats.

He was conscious and oriented, with a patent airway and no focal neurological deficit. Oxygen saturation was 94% on room air without respiratory distress. Blood pressure was 118/83 mmHg, and heart rate was approximately 101 beats per minute with an irregular rhythm. Respiratory examination revealed bilateral basal crackles. Bilateral pitting edema was present. These findings suggested that the patient was clinically stable but not physiologically reassuring.

The left lower limb was cold and pale, with a prolonged capillary refill time of five seconds and absent palpable popliteal, dorsalis pedis, and posterior tibial pulses. Motor and sensory function were preserved, and there was no rest pain. No bedside arterial or venous Doppler signal assessment was documented in the available day one records. The vascular presentation was therefore clinically compatible with Rutherford I-IIa acute limb ischemia. In the absence of documented Doppler signals, a definitive retrospective distinction between Rutherford I and IIa cannot be made. At the same time, the ABCDE assessment showed that the patient was clinically stable but not physiologically simple (Table 1).

**Table 1:** Initial emergency assessment of vascular threat and systemic warning signs Interpretive note: This table highlights the parallel assessment of immediate limb threat and systemic physiological warning signs at the index presentation.

Domain	Finding	Immediate interpretation	Emergency question
Airway/disability	Conscious, speaking, and oriented; no focal neurological deficit	No immediate airway or neurological threat	Is there a competing neurological emergency?
Breathing	Oxygen saturation 94% on room air; no dyspnea; bilateral basal crackles	No respiratory failure, but possible pulmonary congestion	Is the absence of dyspnea falsely reassuring?
Circulation: Systemic	Blood pressure 118/83 mmHg; heart rate approximately 101 beats per minute; irregular rhythm; bilateral pitting edema	No shock, but possible cardiac decompensation or low-output physiology	Is this a low-reserve circulatory state?
Circulation: Limb	Cold and pale left lower limb; capillary refill time five seconds; absent palpable popliteal and distal pulses; preserved motor and sensory function; no rest pain	Clinically compatible with Rutherford I-IIa acute limb ischemia; definitive distinction limited by absent Doppler documentation	Is the limb viable, threatened, or irreversibly damaged?
Exposure/goals	Advanced metastatic cancer; recent chemotherapy; autonomous at baseline; no documented limitation of care	High competing risk within an active oncological pathway	How should technical feasibility be balanced against physiological benefit?

Investigations

On day one, electrocardiography demonstrated atrial fibrillation with a moderate ventricular response, left axis deviation, peripheral low voltage, and no ST-segment elevation (Figure 1). In acute limb ischemia, atrial fibrillation raised both an embolic possibility and a broader hemodynamic question [11-13].

**Figure 1:**
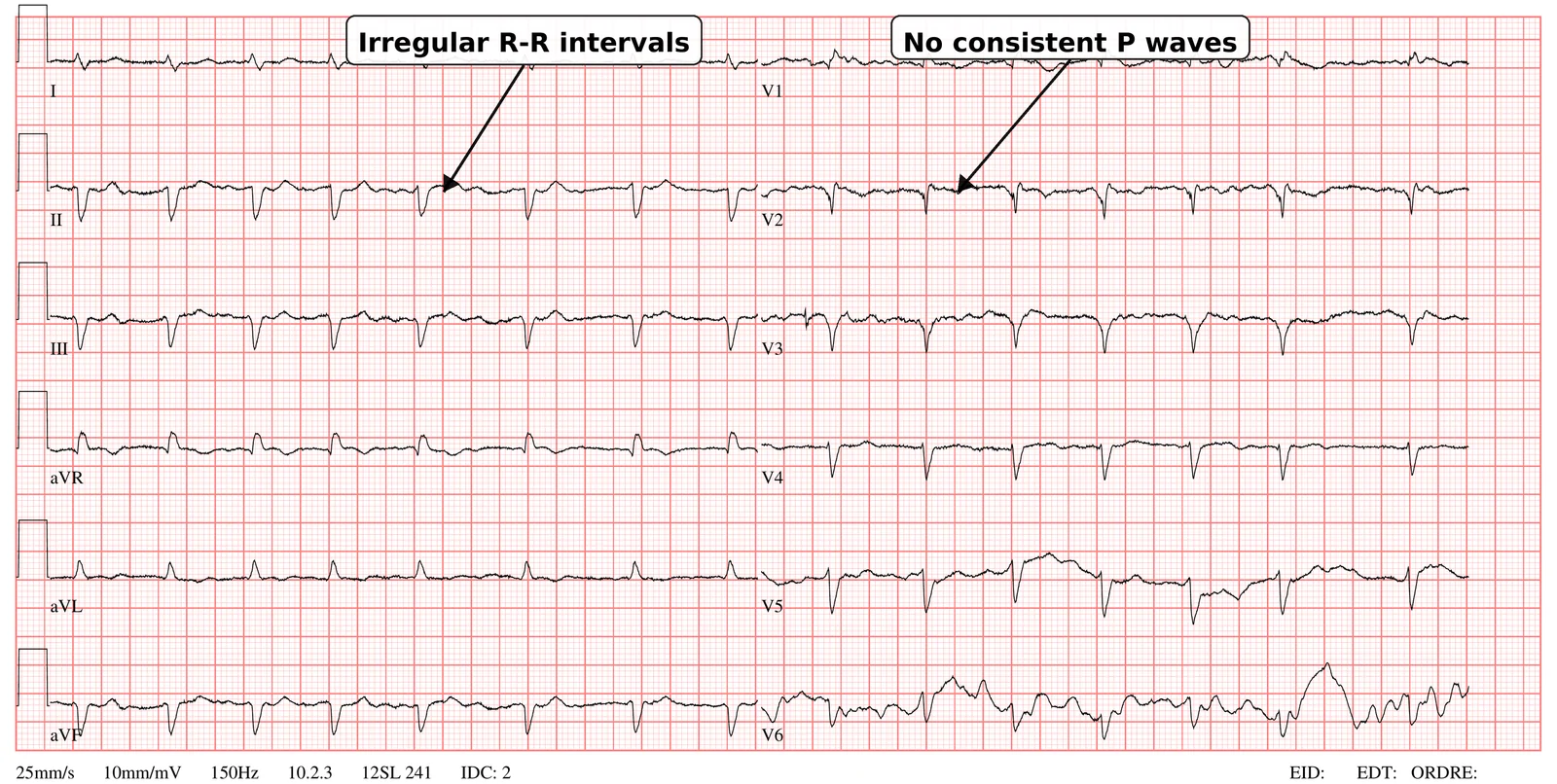
Day one electrocardiogram demonstrating atrial fibrillation An anonymized 12-lead electrocardiogram obtained at the index emergency department presentation shows an irregularly irregular rhythm without consistent P waves, compatible with atrial fibrillation. Annotations highlight the irregular R-R intervals and absence of consistent P waves.

Arterial blood gas analysis showed mild respiratory alkalosis without metabolic acidosis. Lactate was 1.0 mmol/L (0.5-2.2 mmol/L). Potassium was 2.9 mmol/L (3.5-5.1 mmol/L), a relevant abnormality in a patient with atrial fibrillation and structural heart disease. Laboratory tests showed marked inflammation, with a C-reactive protein level of 190 mg/L (<5 mg/L); elevated high-sensitivity troponin T at 142 ng/L, decreasing to 123 ng/L (<14 ng/L); N-terminal pro-B-type natriuretic peptide above 35,000 pg/mL (<450 pg/mL for adults aged 75 years or older); creatine kinase at 527 U/L (39-308 U/L); lactate dehydrogenase at 414 U/L (135-225 U/L); elevated total bilirubin; and preserved renal function. Urinalysis later documented Escherichia coli bacteriuria, while blood cultures remained negative. The troponin pattern and absence of typical symptoms or electrocardiographic changes argued against type 1 myocardial infarction but supported myocardial injury or type 2 myocardial infarction in a multimorbid context [12]. Chest radiography showed enlargement of the cardiomediastinal silhouette, a left subclavian port-a-cath, and a small right pleural effusion without obvious pulmonary consolidation.

Computed tomography angiography (CTA) on day one demonstrated greater than 75% stenosis of the distal left superficial femoral artery at the adductor canal, poor opacification of the left popliteal artery, and limited distal runoff (Figure 2; panels A, B, and D). The popliteal and crural arteries were difficult to assess because of artifact and radiological concern for low cardiac output; this represented an imaging interpretation rather than a hemodynamically confirmed diagnosis. The same examination also demonstrated large bilateral pleural effusions on the lower thoracic slices (Figure 2; panel C). During the subsequent endovascular procedure, the lesion was described as subocclusive distal superficial femoral artery stenosis with long popliteal occlusion, very slow flow, and predominantly distal collateralization. Thus, imaging obtained for the threatened limb also provided evidence that the clinical problem might not be purely peripheral.

**Figure 2:**
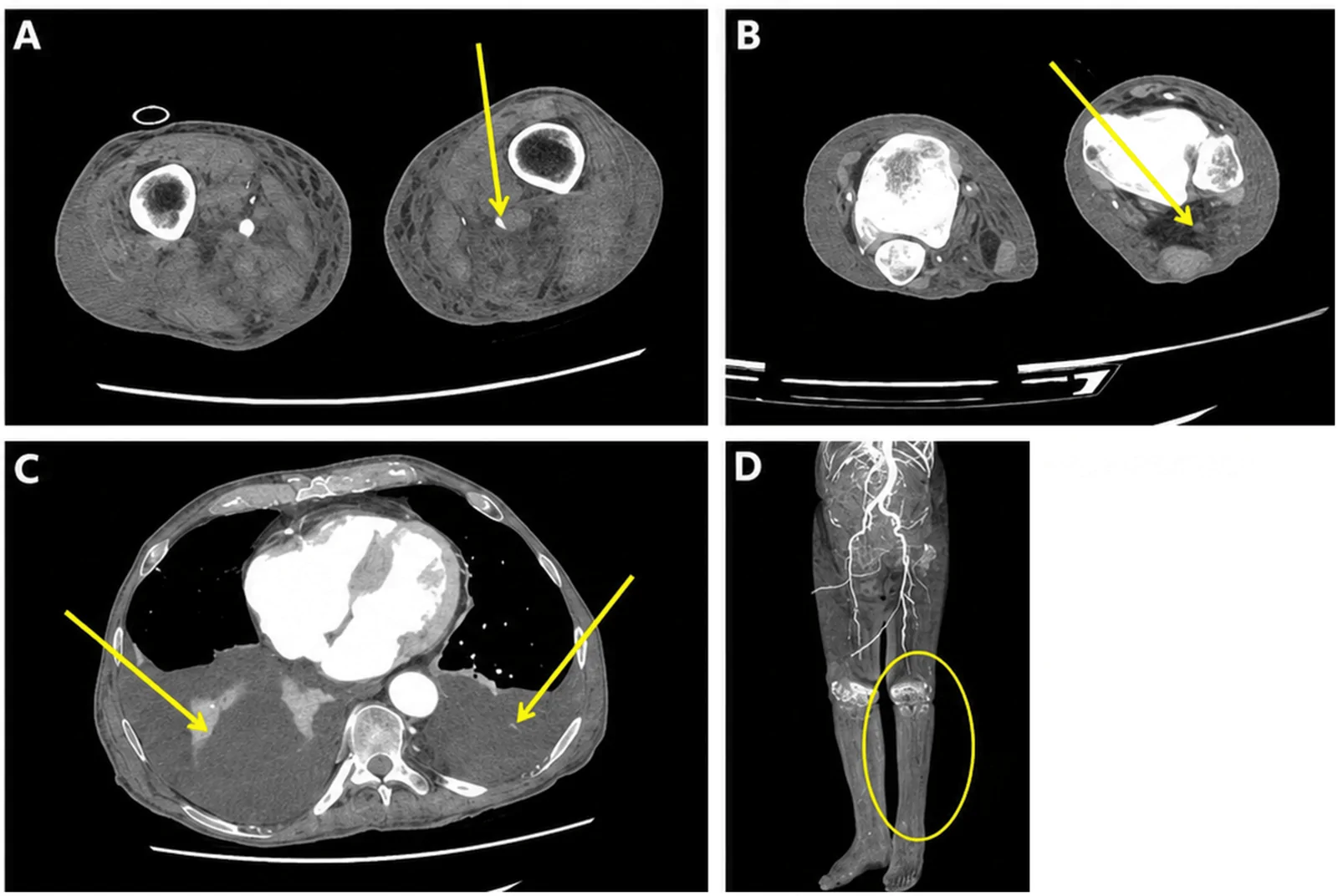
Day one CTA findings A: Axial CTA showing severe stenosis of the distal left superficial femoral artery at the adductor canal (arrow); B: Axial CTA showing poor opacification of the left popliteal artery (arrow); C: Axial CTA through the lower thorax demonstrating large bilateral pleural effusions (arrows); D: Volume-rendered CTA runoff demonstrating limited distal arterial opacification of the left lower limb (circle) CTA: Computed tomography angiography

Differential diagnosis

Overall, the imaging and procedural findings favored acute limb ischemia superimposed on chronic peripheral arterial disease rather than a purely embolic event. Severe focal superficial femoral artery disease, a long popliteal occlusion, and distal collateralization supported an acute-on-chronic ischemic process. Active metastatic adenocarcinoma, recent chemotherapy, and systemic inflammation increased thrombotic risk [4,5]. New atrial fibrillation remained a potential embolic contributor. Suspected low-flow physiology may also have worsened distal perfusion across previously compensated arterial disease.

The cardiovascular abnormalities prompted concern for possible decompensated heart failure and/or suspected low-output physiology in the setting of new atrial fibrillation and hypertrophic cardiomyopathy [11,13]. These remained working hypotheses rather than confirmed diagnoses because no contemporaneous echocardiography or invasive hemodynamic assessment was obtained. Myocardial injury or type 2 myocardial infarction was also plausible, driven by tachyarrhythmia, systemic inflammation, anemia, possible low flow, and malignancy [12]. Type 1 myocardial infarction was less likely because there was no chest pain, no ST-segment elevation, and no rising troponin pattern. The FOLFOX-related cardiotoxicity was considered as a secondary cardio-oncology possibility, since fluoropyrimidines can be associated with coronary vasospasm, arrhythmias, myocarditis, or heart failure, but the presentation was not typical enough to make this the leading explanation [14,15].

Treatment

After multidisciplinary discussion involving emergency medicine and the vascular team, endovascular revascularization was pursued for limb salvage and symptom relief. The decision was defensible based on the information available at the time: the patient was still autonomous, receiving active oncological treatment, had no documented therapeutic limitation, was not in shock, had preserved renal function, and had a clinically significant acute ischemic presentation within the Rutherford I-IIa spectrum, together with severe arterial disease on imaging. Bedside Doppler signals were not documented, preventing definitive retrospective categorization.

On day two, he underwent percutaneous left femoropopliteal angioplasty with placement of a 6 mm x 60 mm covered stent across the distal superficial femoral artery lesion. The available procedural documentation described a satisfactory technical result at the treated lesion but also recorded no meaningful distal runoff ('distalité borgne'), indicating severely compromised distal outflow. A long popliteal occlusion was documented angiographically; the available procedure note does not describe separate treatment or successful recanalization of this segment.

Therapeutic-dose anticoagulation had been initiated during the day one emergency department assessment because of the newly diagnosed atrial fibrillation and possible embolic contribution. This therapeutic anticoagulation was not maintained during the subsequent vascular surgery admission. At discharge on day three, the prescribed antithrombotic regimen consisted of enoxaparin 40 mg subcutaneously once daily for 10 days and aspirin 160 mg once daily; pentoxifylline was also prescribed. The available medical record does not document the rationale for changing from therapeutic anticoagulation to prophylactic-dose enoxaparin.

The postoperative course was described as uncomplicated and clinically favorable in the discharge documentation. No postoperative arterial Doppler study or repeat vascular imaging was documented in the records available for this report, and no quantified assessment of distal arterial patency before discharge was identified. A cardiology/internal medicine assessment had been requested on day one because of the new atrial fibrillation and broader cardiovascular abnormalities, but this assessment was not completed before discharge on day three. This point is not presented as criticism of an individual or service but as the organizational pivot of the case: once the patient entered a vascular pathway, the systemic problem became harder to keep visible. A vascular surgery follow-up visit was planned approximately one month after discharge.

Outcome and follow-up

On day five, the patient returned with worsening left lower limb ischemia. The family reported violet discoloration below the knee. Examination showed a cold foot, absent distal pulses, and necrotic toes, without documented motor or sensory deficit. He remained hemodynamically stable. Repeat arterial blood gas analysis again showed a lactate level of 1.2 mmol/L (0.5-2.2 mmol/L) despite distal necrosis, with persistent alkalosis and hypokalemia. This reinforced an important emergency medicine point: a normal systemic lactate does not exclude severe localized limb ischemia or distal tissue necrosis.

No repeat vascular imaging was identified in the available day five records to establish the mechanism of recurrent ischemia. Potential contributors included persistent distal outflow obstruction, early thrombosis or reocclusion, progression of an acute-on-chronic ischemic process, cancer-associated thrombosis, an embolic contribution from newly diagnosed atrial fibrillation, and suspected systemic low-flow physiology.

Given the absence of clinical improvement and lack of further feasible revascularization, vascular surgery considered that amputation would not offer meaningful benefit in the context of advanced malignancy and rapid systemic decline. Chemotherapy was stopped because of uncontrolled necrosis and septic risk. The patient rapidly lost autonomy, and a palliative approach was formally adopted on day 13. He died in a palliative care unit on day 58. No postmortem examination was performed. The clinical course from the index emergency presentation through revascularization, recurrent ischemia, and transition to palliative care is summarized in Table 2.

**Table 2:** Clinical timeline from index emergency presentation to palliative outcome Interpretive note: Day one denotes the index emergency department presentation. The timeline emphasizes the distinction between technical treatment of the focal arterial lesion and the subsequent clinical course.

Day no.	Event	Interpretation
1	Emergency department presentation with a cold, pale left lower limb, capillary refill time of five seconds, absent palpable popliteal and distal pulses, and preserved motor and sensory function; no bedside Doppler signals documented.	Clinically compatible with Rutherford I-IIa acute limb ischemia, with concurrent systemic cardiovascular warning signs.
2	Percutaneous femoropopliteal angioplasty with a 6 mm x 60 mm covered stent across the distal superficial femoral artery lesion; no meaningful distal runoff documented.	Technical success at the treated superficial femoral artery lesion with severely compromised distal outflow.
3	Discharged on enoxaparin 40 mg subcutaneously once daily for 10 days and aspirin 160 mg once daily; therapeutic anticoagulation was not continued. No postoperative arterial Doppler or repeat vascular imaging was documented.	The systemic cardiovascular problem and objective distal arterial patency remained incompletely characterized.
5	Return with cold foot and toe necrosis; lactate remained normal; no repeat vascular imaging was available.	Recurrent limb ischemia with uncertain mechanism.
13	Formal transition to a palliative approach after chemotherapy was stopped and autonomy declined.	Palliative goals established after clinical failure and rapid systemic decline.
58	Death in a palliative care unit.	Terminal outcome after rapid systemic and functional decline.

## Discussion

A correct diagnosis can narrow reasoning

The central lesson of this case is that the cold limb was not a misleading diagnosis. It was correct, real, and time-sensitive. The problem was more subtle: a correct specialized diagnosis can still become the endpoint of reasoning when the patient's global physiology has not yet been integrated.

This pattern is best understood through the diagnostic reasoning literature. Anchoring describes the tendency to remain attached to an early impression; premature closure describes stopping the diagnostic process before adequate verification; diagnostic momentum describes how a label can gather force as it passes between clinicians; and search satisficing describes ending the search after finding one plausible abnormality [6-8]. In this case, acute limb ischemia did not cause a missed vascular diagnosis. It risked causing diagnostic narrowing after the vascular diagnosis had been made. The emergency physician's task is therefore not only to identify the correct specialist pathway. It is also to prevent that pathway from erasing the rest of the patient.

Why revascularization remained defensible

The unfavorable outcome should not lead to hindsight bias. At presentation, the patient lived at home, was undergoing active cancer treatment, was not in shock, had preserved renal function, and had no documented limitation of care. The limb was cold and pale, with a capillary refill time of five seconds and absent palpable popliteal and distal pulses, while motor and sensory function were preserved and there was no rest pain. These findings were clinically compatible with Rutherford I-IIa acute limb ischemia. Because bedside arterial and venous Doppler signals were not documented, a definitive retrospective distinction between Rutherford I and IIa cannot be made. For context, the Rutherford acute limb ischemia categories are summarized in Table 3 [1,2]. A vascular opinion and an attempt at revascularization were therefore reasonable based on the information available at the time.

**Table 3:** Rutherford classification of acute limb ischemia Summary based on the Rutherford acute limb ischemia criteria presented in references [1,2]. In this case, motor and sensory function were preserved, and there was no rest pain, but bedside arterial and venous Doppler signals were not documented. The available data therefore support a Rutherford I-IIa spectrum but do not permit a definitive retrospective distinction between these categories.

Category	Status/prognosis	Sensory loss	Muscle weakness	Arterial Doppler	Venous Doppler
I	Viable; not immediately threatened	None	None	Audible	Audible
IIa	Marginally threatened; salvageable if promptly treated	None or minimal (toes)	None	Often inaudible	Audible
IIb	Immediately threatened; salvageable with immediate revascularization	More than toes; usually associated with rest pain	Mild or moderate	Inaudible	Audible
III	Irreversible; major tissue loss or permanent nerve damage inevitable	Profound; anesthetic	Profound; paralysis or rigor	Inaudible	Inaudible

The lesson is not that revascularization should be withheld solely because of malignancy. Evidence suggests that limb salvage can remain achievable in selected cancer patients with acute limb ischemia, even though mortality is high [16,17]. The important point is that technical feasibility should be discussed alongside distal perfusion, expected functional recovery, cardiac reserve, prognosis, and goals of care. In this case, technical success at the treated superficial femoral artery lesion coexisted with severely compromised distal outflow, and clinical recovery did not follow.

Why recurrent ischemia remained mechanistically uncertain

The mechanism of recurrent ischemia cannot be established retrospectively. Persistent distal outflow obstruction was already documented during the intervention. Other potential contributors include early thrombosis or reocclusion, progression of acute-on-chronic peripheral arterial disease, cancer-associated thrombosis, an embolic contribution from newly diagnosed atrial fibrillation, and suspected systemic low-flow physiology. No repeat vascular imaging was available to distinguish among these mechanisms.

Vascular-plus-physiology cross-check, a practical educational implication

This single case cannot establish a new protocol. We propose a brief vascular-plus-physiology cross-check as an educational framework for emergency medicine; it has not been prospectively evaluated or validated as a clinical decision algorithm. In older or multimorbid patients, or patients receiving active oncological treatment, with acute limb ischemia, the cross-check is intended to run in parallel with and not delay the vascular pathway. Its purpose is to keep both problems explicit: the threatened limb and the potentially threatened systemic physiology (Figure 3).

**Figure 3:**
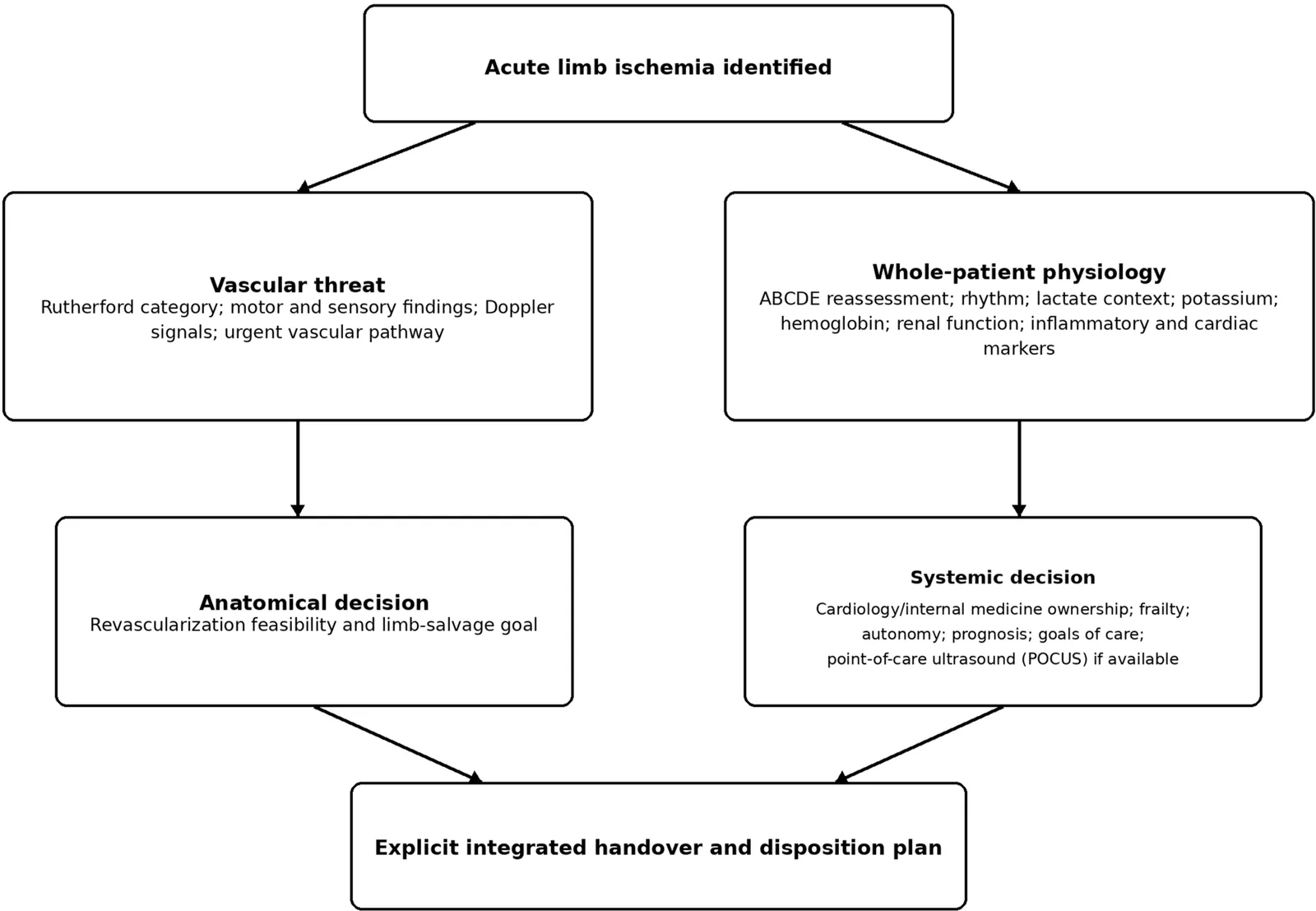
Proposed emergency medicine vascular-plus-physiology cross-check This author-proposed educational framework illustrates a parallel approach to vascular threat and whole-patient physiology; it has not been prospectively evaluated or validated as a clinical decision algorithm. The vascular diagnosis should activate the specialty pathway while preserving explicit attention to whole-patient physiology, ownership, POCUS if available, and goals of care. ABCDE: Airway, breathing, circulation, disability, exposure; POCUS: Point-of-care ultrasound

Such a cross-check can be brief: Rutherford category and limb Doppler signals; ABCDE reassessment; electrocardiographic rhythm; lactate interpretation without false reassurance; potassium, hemoglobin, renal function, inflammatory markers, and cardiac biomarkers; review of CTA for systemic clues such as pleural effusions or suspected low flow; baseline autonomy and goals of care; and explicit ownership of the nonvascular problem after the vascular decision. When systemic red flags are present, handover should be explicit enough to survive specialty transfer: the vascular question, the systemic physiological concern, and responsibility for follow-up should each be named. This separates the limb-salvage decision from the disposition and global-care decision.

Potential role of point-of-care ultrasound (POCUS)

At the time of the case, the emergency physician was not yet trained in focused cardiac ultrasound. It would therefore be unfair to present the absence of POCUS as a retrospective standard-of-care failure. The case instead illustrates why POCUS may be useful in similar future presentations.

Focused lung ultrasound could have looked for B-lines and pleural effusions [18]. Focused cardiac ultrasound could have assessed gross left ventricular systolic function, marked hypertrophy, chamber dilation, right ventricular dilation, pericardial effusion, and gross signs of low-output physiology [19,20]. Inferior vena cava assessment could have contributed cautiously to the volume-status discussion. Peripheral vascular Doppler could have documented asymmetry or absence of distal arterial signals [1,2]. The value would have been partly diagnostic but also communicational. In a patient moving rapidly through a vascular pathway, POCUS images may make systemic physiology visible and shareable, strengthening the argument for early cardiology/internal medicine involvement and avoiding a purely anatomical definition of success.

Limitations

This is a single case and cannot determine which patients with malignancy-associated acute limb ischemia should or should not undergo revascularization. The retrospective nature of the analysis creates a risk of hindsight bias. Bedside arterial and venous Doppler signals were not documented at the day one presentation, limiting retrospective confirmation of the Rutherford category. No echocardiography was performed during the episode, and the most recent formal echocardiogram had been performed approximately 15 years earlier, limiting certainty about contemporary cardiac function; possible decompensated heart failure and suspected low-output physiology therefore remained unconfirmed. No postoperative arterial Doppler study or repeat vascular imaging before discharge was identified in the available records, and no repeat vascular imaging was available at the day five recurrence, limiting assessment of patency and mechanism. The available record also did not document the rationale for changing from therapeutic anticoagulation to prophylactic-dose enoxaparin. No postmortem examination was performed, so the exact contribution of thrombosis, embolism, persistent distal outflow obstruction, systemic low-flow physiology, infection, reperfusion injury, and cancer progression cannot be proven.

These uncertainties are part of the educational value of the case. Emergency physicians often make urgent decisions before the mechanism is fully defined. The proposed practical lesson is therefore not a new diagnostic certainty but a safer way of maintaining physiological integration in a patient with competing emergencies.

## Conclusions

Acute limb ischemia can be both a real emergency and a diagnostic attractor. In older patients with advanced malignancy and multimorbidity, the threatened limb should be assessed in parallel with systemic physiological reserve. Normal blood pressure and normal systemic lactate do not exclude severe localized ischemia or a clinically important low-reserve cardiovascular state.

New atrial fibrillation, basal crackles and peripheral edema, markedly elevated N-terminal pro-B-type natriuretic peptide, troponin elevation, pleural effusions, and radiological concern for low flow should prompt explicit consideration of possible cardiac decompensation and early cardiology/internal medicine involvement even when a vascular pathway is activated. In this case, neither decompensated heart failure nor low-output physiology was definitively confirmed because contemporaneous echocardiographic and hemodynamic assessment was unavailable. The vascular-plus-physiology cross-check proposed here is an educational suggestion rather than a validated clinical algorithm. Technical success at a focal arterial lesion should not be equated with restoration of distal perfusion, systemic recovery, functional benefit, or alignment with patient goals.
